# Use of NTCP metrics to identify unbalanced levels of dose trade‐off between left lung and heart in lung cancer radiotherapy

**DOI:** 10.1002/acm2.70207

**Published:** 2025-08-21

**Authors:** Georgios Komisopoulos, Panayiotis Mavroidis, Fotini Simopoulou, George Kyrgias, Kiran Pant, Yiannis Roussakis, Kyriaki Theodorou

**Affiliations:** ^1^ Department of Medical Physics Medical School University of Thessaly Larissa Greece; ^2^ Department of Radiation Oncology University of North Carolina at Chapel Hill North Carolina USA; ^3^ Department of Radiation Oncology IASO Thessaly Medical Center Larissa Greece; ^4^ Department of Medical Physics German Oncology Center University Hospital of the European University Limassol Cyprus

**Keywords:** dose trade‐off, heart valve dysfunction, lung cancer radiotherapy, normal tissue complication probability, radiation pneumonitis

## Abstract

**Introduction:**

The aim of this study is to determine the optimal level of dose trade‐off between left lung and heart during plan optimization in lung cancer radiotherapy, and also to examine the impact of planning target volume (PTV) size in reducing the overall risk of toxicity of lung and heart.

**Methods:**

This work includes 20 left‐sided lung cancer patients and for each of them two VMAT plans (dosimetrically and radiobiologically optimized) were developed. In all the plans, a dose of 70 Gy in 35 fractions was prescribed to PTV. Each plan was evaluated in terms of PTV coverage, mean lung dose, lung‐V_20_, mean heart dose, and heart‐V_5_. The normal tissue control probability (NTCP) values of radiation pneumonitis and heart valve dysfunction were calculated using the Relative Seriality model.

**Results:**

The patients with PTV < 300 cm^3^ had a left lung NTCP of 70.7 ± 14.6% and a heart NTCP of 11.5 ± 5.5%, whereas for the patients with PTV > 300 cm^3^, the corresponding NTCP values were 86.0 ± 9.5% for left lung and 13.7 ± 4.9% for heart, respectively. After the NTCP‐based optimization, for the patients with PTV < 300 cm^3^, the corresponding lung NTCP values had a decrease of 12.8% and the heart NTCP values had an increase of 2.7%, whereas for the patients with PTV > 300 cm^3^, the lung NTCP values had a decrease of 6.5% and the heart NTCP values had an increase of 3.9%.

**Conclusion:**

Our results indicate that there is a systematic unbalance in dose trade‐off between left lung and heart, which varies based on the PTV size. NTCP metrics were found useful in identifying this issue.

## 1 INTRODUCTION

The primary goal of treatment plan optimization is to find a beam configuration that will produce the optimal dose distribution in terms of target coverage and normal tissue sparing. However, in most cases, it is challenging to determine the optimal dose trade‐offs between the primary organs at risk (OAR). Different techniques have been proposed to achieve this goal. One such technique involves the use of dose constraints (clinical goals) that have been found by clinical trials to reduce the risk of given radiation induced symptoms after radiotherapy (post‐RT). Another technique employes optimization tools, such as PlanIQ (Sun Nuclear Corp., Melbourne, Florida, USA), which determine a range of feasible minimum dose‐volume points/constraints for every OAR considering the anatomical characteristics of the individual patient (e.g., location and shape of the targets and OARs) and the prescription target doses.^1^, ^2^ In both of those approaches, an optimizer tries to find the minimum of a cost function, which incorporates the desired doses and constraints using different weights. Those weights indicate the priorities by which the goals of the optimization will be satisfied. Although those techniques have helped to reduce the inter‐planner variability in identifying feasible plans of similar qualities, they still have not determined a way to identify the optimal or a desired level of dose trade‐offs between the different OARs. In lung cancer radiotherapy, heart and lung are usually the primary OARs that need to be spared to reduce the corresponding risks of post‐RT complications.

A two‐fold increase in cardiovascular death has been reported in patients who have undergone radiotherapy.^3^, ^4^, ^5^ Radiation dose has been found to be a major risk factor for subsequent development of cardiovascular disease. More specifically, the risk of valvular heart disease (VHD) shows a linear increase with dose above 30 Gy.^6^ Also, thoracic radiation has been associated with valvulopathy after radiotherapy (26%–30% at 10 years and 60% at 20 years post‐RT).^3^, ^4^ These patients have a 9.2‐fold increased risk of requiring surgery for their valvulopathy compared to non‐irradiated subjects.^7^ Although late cardiac toxicity is one of the most severe radiation related side effects, the relevant data are still limited. Modeling such a toxicity is difficult due to the lack of long‐term results.^8^, ^9^, ^10^, ^11^ The QUANTEC Reports have determined heart parameters for the Lyman‐Kutcher‐Burman (LKB) and Relative Seriality (RS) normal tissue complication probability (NTCP) models.^12^ The clinical endpoints that were examined were cardiac perfusion defects, very delayed cardiac mortality, and pericarditis/pericardial effusion. Another important side effect of thoracic radiation therapy is the manifestation of valvular abnormalities.^13^ However, until now, very few studies have tried to model this specific radiation‐induced heart complication.

Radiation pneumonitis (RP) is the most common and clinically relevant toxicity of lung or breast cancer radiation therapy. About 30% (or 83% for RP grade ≥ 1) of patients treated with conventionally fractionated radiation therapy for locally advanced lung cancer and 10% of patients treated with stereotactic body radiation therapy (SBRT) for lung tumors report a grade ≥ 2 of this toxicity.^14^, ^15^, ^16^, ^17^ Mean lung dose and lung V_20Gy_ are the dose metrics that have been associated with the development of RP post‐RT. Historically, fatal RP has an incidence of about 2% and 1% after conventional radiotherapy and SBRT, respectively.^18^ One of the difficulties of modeling the dose‐response relationship of RP is that different dose calculation algorithms (DCAs) predict different dose distributions for the same treatment.^19^ Kavousi and colleagues used the LKB model to determine the model parameter values for different DCAs.^20^ Those DCAs were the pencil beam convolution (PBC), collapsed cone (CC), and Monte Carlo (MC), which have been implemented in Monaco, the analytical anisotropic algorithm (AAA), which has been implemented in Eclipse, and finally the superposition and Clarkson algorithms, which have been implemented in PCRT3D treatment planning systems. Those DCAs were used in the calculation of lung dose, which subsequently resulted in > 15% deviation in NTCP values for the same model parameters.

In the present study, NTCP model parameters that correspond to lung and heart related symptoms of similar severity were used. This is fundamental in such kind of planning studies because the NTCP metrics that are used in dose optimization should reflect risk rates of symptoms that are equivalent in severity otherwise different weighting factors should be applied that would correspond to the clinically desired NTCP ratios between the examined OARs.

## 2 METHODS

### 2.1 Extended historical cohort to establish the case of unbalanced dose trade‐offs

To determine whether there is a systematic unbalance in dose trade‐off (over‐sparing one organ at the expense of another) between left lung and heart, the clinical dosimetric data of 61 consecutive lung cancer patients, who were treated with intensity‐modulated radiation therapy (IMRT) were retrospectively analyzed. For each patient, the dose volume histograms (DVHs) of lung [minus planning target volume (PTV)] and heart were exported together with the volume of PTV and the minimum distance between PTV and heart. Those DVHs were used to calculate the corresponding NTCP values of RP and heart valve dysfunction (RVD) using the Relative Seriality model.

### 2.2 Patient selection for re‐optimization

The dosimetric data of 20 consecutive patients, who were treated for lung cancer with volumetric modulated arc therapy (VMAT), were retrospectively collected. In total, 20 VMAT plans (dosimetrically optimized) and 20 VMAT replans (NTCP‐optimized) were created. Specifically, the initial (dosimetrically optimized) plan of each patient was re‐optimized using different levels of dose trade‐off between left lung and heart while maintaining the PTV coverage the same. The NTCPs of left lung and heart were calculated for all the different levels of dose trade‐off and the plan and the most balanced set of NTCPs was selected as optimal. All the patients were scanned in 3 mm thickness slices in the supine position with both arms above their heads using a 16‐detector array computed tomography (CT) scanner (Toshiba Aquilion 16). Scanning was performed with free breathing. The same radiation oncologist contoured the clinical target volume (CTV) and PTV. Furthermore, heart, lungs (ipsilateral, contralateral, and paired), esophagus, and spinal cord were considered as OARs. The PTV volumes of the 20 patients, ranged between 62.9  and 626.5 cm^3^ (the median PTV volume was 300 cm^3^).

### 2.3 VMAT plans

**FIGURE 1:**
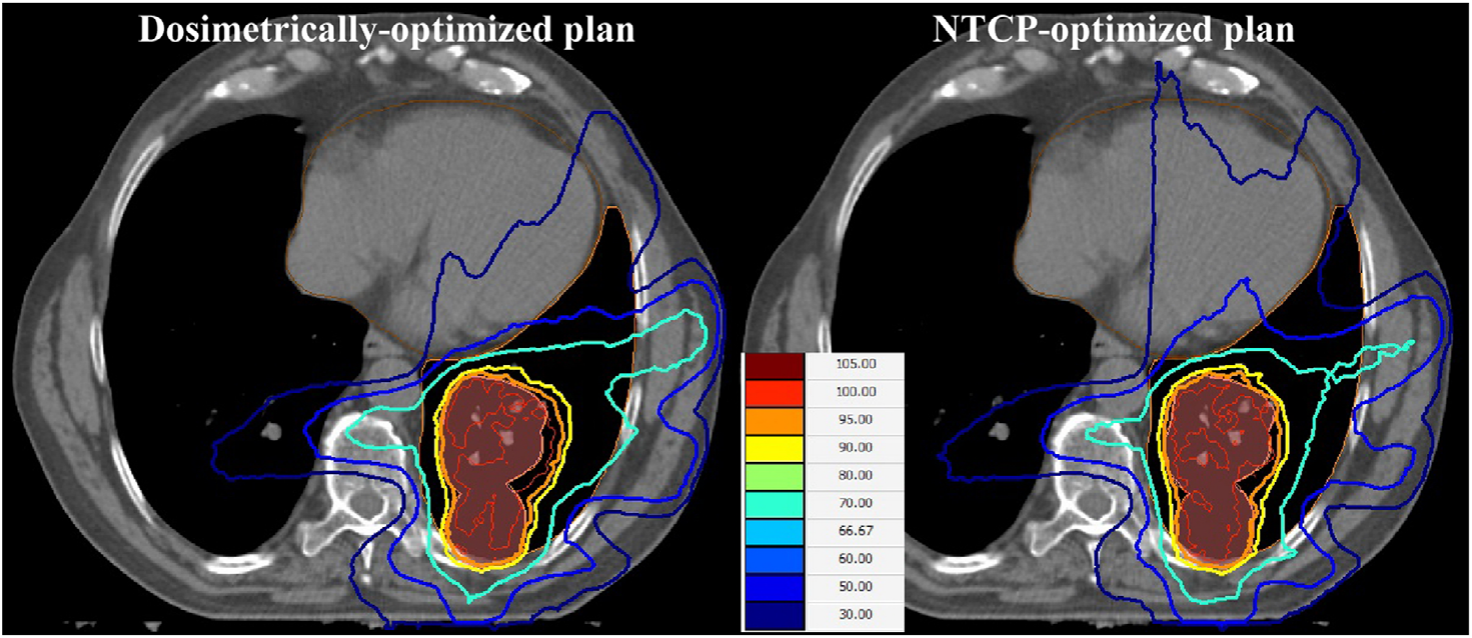
Dose distributions of the dosimetrically optimized (left) and NTCP‐optimized (right) plans in the form of isodose lines for one of the patients. The colormap of the isodose lines is also shown.

Two VMAT plans were created for each patient using one Arc (180°–220°) depending on PTV size and location. All the plans used 6 MV photon beams and were optimized with 200 maximum number of control points per beam and 1 cm minimum segment width. The fractionation scheme of those plans was 70 Gy in 35 fractions. The dosimetrically‐ and NTCP‐optimized plans of a representative patient are shown in Figure 1. For all the plans, the Monaco (Elekta AB, Stockholm, Sweden) treatment planning system was used together with the collapsed cone as the DCA.

### 2.4 Treatment planning endpoints

Treatment plans were prescribed as follows: V_70_ to the whole PTV should be at least 95%. Additionally, the mean dose of PTV was prescribed to 70 Gy. Measured data for plan comparison included:

- Coverage of PTV.
- Overall maximum plan dose.
- Mean lung dose (MLD) and V20 to left lung (minus PTV).
- Mean heart dose (MHD) and V5 to heart.
- Mean dose and V20 to paired‐lungs (minus PTV).
- Maximum dose to spinal cord.
- V60 to esophagus.

**FIGURE 2:**
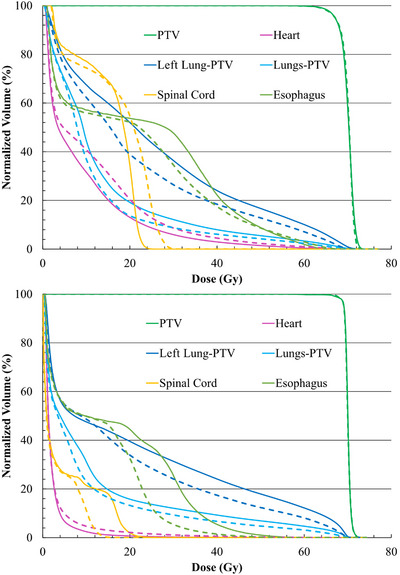
Dose volume histograms (DVHs) of PTV, heart, left lung (minus PTV), paired‐lungs (minus PTV), esophagus, and spinal cord for the dosimetrically and NTCP‐optimized plans for two patients with PTV > 300 cm^3^ (top) and PTV < 300 cm^3^ (bottom), respectively. The solid and dashed lines correspond to the dosimetrically and NTCP‐optimized plans, respectively.

A summary of the prescribed doses and dose constraints to the OARs is shown in Table 1.^21^, ^22^, ^23^, ^24^ For two representative patients with PTV > 300 and < 300 cm^3^, the DVHs of the targets and OARs for the dosimetrically‐ and NTCP‐optimized plans are shown in Figure 2. In the process of re‐optimizing the initial dosimetrically optimized plans, the intention was to achieve the same PTV coverage and level of sparing in the rest of the organs at risk.

**TABLE 1:** Summary of the dose constraints and clinical goals for the target and organs at risk used in treatment plan optimization.

Dose constraints	Value
PTV D_95%_	≥ 70 Gy
Mean heart dose (MHD)	≤ 20 Gy [18]
Heart V_45_	≤ 67% [18]
Heart V_5_ (*)	≤ 50% [19]
Left lung mean dose (*)	≤ 22 Gy [20]
Left lung V_20_	≤ 52% [20]
Mean paired‐lungs dose	≤ 20 Gy [21]
Paired‐lungs V_20_	≤ 30% [21]
Maximum spinal cord dose	≤ 50 Gy
Esophagus V_60_	≤ 50%

(*) Additional optional dose constraints.

### 2.5 Radiobiological analysis and statistical methods

The dosimetric and radiobiological analysis was based on the DVHs of the different organs per patient. All the relevant metrics were derived from those DVHs (e.g., average doses, standard deviations, range).^25^, ^26^ For the radiobiological analysis, the Poisson and Relative Seriality models were used to calculate the expected tumor control probability (TCP) and NTCP values of the PTV and OARs, respectively.^25^, ^26^, ^27^, ^28^, ^29^, ^30^, ^31^, ^32^, ^33^, ^34^

Ideally, patient‐reported outcome (PRO) of the Common Toxicity Criteria for Adverse Events (CTCAE) or physician‐based CTCAE scores at baseline (pre‐RT) and at 6‐ or 12 months post‐RT should be used for a solid NTCP modeling analysis, which would provide model parameter values for plan optimization studies. However, other NTCP modeling studies, which used other scores for determining the model parameters of moderate cardiac endpoints, were found to be more suitable for the purpose of this analysis.

Regarding the model parameter values of left lung and heart, they were taken from the literature. More specifically, Cella et al.^32^ determined the parameter values of the LKB and RS NTCP models for the clinical endpoint of heart valve dysfunction (RVD). For RP, Kavousi et al.^19^ used the data reported by Hedin and Bäck, Rancati et al.^35^, ^36^, ^37^ to calculate the parameter values of the LKB model for different DCAs. The parameter values of the RS model were calculated through fitting the LKB‐based dose‐response curve for the Collapse Cone algorithm. Those model parameter values were derived for the endpoint of RP grade ≥1. Table 2 presents the model parameters of PTV, left lung, and heart that were used to calculate the TCP and NTCP metrics. The results were analyzed using a paired *t*‐test. A resultant *p*‐value of < 0.05 implied a statistically significant difference.

**TABLE 2:** Summary of the relative seriality model parameter values for the target, heart, and lung.

Organ/parameters	*D*_50_ (Gy)	*γ*	*s*	*α/β* (Gy)
PTV^31^	49.2	1.0	—	10.0
Heart^32^	32.4 (22.7, 48.5)	0.42 (0.24, 0.62)	1.0 (0.0–1.0)	3.0
Lung (single)^20^	15.55 (15.46, 15.64)	1.2 (1.1, 1.3)	0.05	3.0

*D*_50_ is the 50% response dose, γ is the maximum normalized value of the dose‐response gradient, and *s* is the relative seriality parameter, which characterizes the volume dependence of the organ. Those model parameters were derived for the clinical endpoints of heart valve dysfunction (RVD) and radiation pneumonitis (grade ≥ 1) for lung.

## 3 RESULTS

### 3.1 Extended historical cohort to establish the case of unbalanced dose trade‐off

**FIGURE 3:**
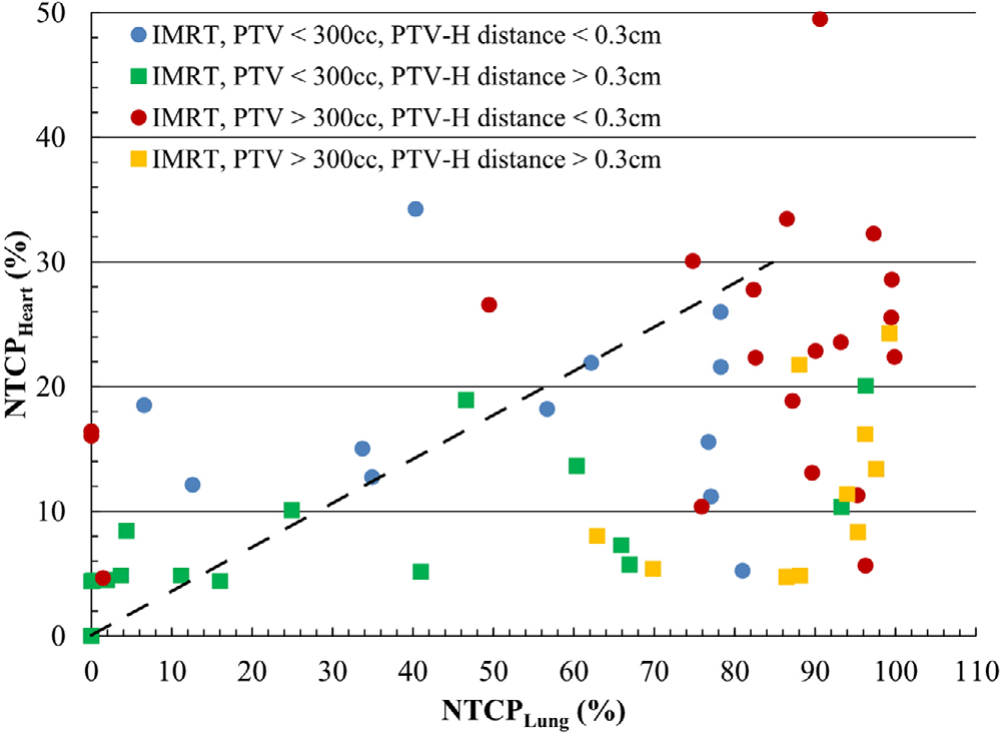
Plot of left lung NTCP vs. heart NTCP for IMRT patients grouped by their PTV size and PTV to heart (PTV‐H) distance. The dashed line indicates the ratio of the clinically reported incidence rates for the examined lung and heart complications.

In this cohort, the median PTV size and median minimum distance between PTV and heart were 300 cm^3^ and 0.3 cm, respectively. To examine if there is a systematic unbalanced dose trade‐off between left lung and heart and whether this depends on PTV volume and PTV‐heart distance, the patient cohort was grouped using the previous values as thresholds. So, the patients with PTV < 300 cm^3^ and PTV‐heart distance < 0.3 cm (mean PTV = 170 cm^3^) had an average lung NTCP value of 53.2%, which is much larger than the average heart NTCP of 17.7%, whereas those with PTV < 300 cm^3^ and PTV‐heart distance > 0.3 cm (mean PTV = 103 cm^3^) had average lung and heart NTCP values of 28.0% and 7.1%, respectively (See Table 3). Similarly, the patients with PTV > 300 cm^3^ and PTV‐heart distance < 0.3 cm (mean PTV = 907 cm^3^) had an average lung NTCP value of 74.6%, which is much larger than the average heart NTCP of 22.1%, whereas those with PTV > 300 cm^3^ and PTV‐heart distance > 0.3 cm (mean PTV = 533 cm^3^) had average lung and heart NTCP values of 87.8% and 11.8%, respectively. A plot of those results is shown in Figure 3, where the dashed diagonal line indicates the ratio of the historical incidence rates (∼85% for grade ≥ 1 RP for lung cancer radiotherapy vs. ∼30% for RVD) of lung and heart. Those results indicate that a systematic unbalanced dose trade‐off between left lung and heart is observed independent of PTV volume and PTV‐heart distance.

**TABLE 3:** Summary of the left lung and heart NTCP values for the different subgroups defined by treatment technique, PTV size, and PTV to heart distance.

Technique	PTV (cm^3^)	PTV‐Heart (cm)	NTCP_L_ (%)	NTCP_H_ (%)	Cutoff values
IMRT	170	0.1	53.2	17.7	PTV<300 cc, PTV‐H<0.3 cm
	103	4.4	28.0	7.1	PTV<300 cc, PTV‐H>0.3 cm
	907	0.0	74.6	22.1	PTV>300 cc, PTV‐H<0.3 cm
	533	1.9	87.8	11.8	PTV>300 cc, PTV‐H>0.3 cm

### 3.2 Dosimetric comparison

The results of the comparison of the dosimetrically‐ and NTCP‐ optimized plans of the 20 patients are presented in Tables 4 and 5, where it is indicated that PTV size has a considerable impact on dose trade‐off between left lung and heart. For this reason, the results were grouped per PTV size using the values of 300 cm^3^ as the cutoff volume (median value in the cohort). For the patients with PTV > 300 cm^3^, the mean PTV coverage (V_70_) values were 96.3% and 96.2% for the dosimetrically‐ and NTCP‐optimized plans, whereas for PTV < 300 cm^3^, those values were 97.6% and 97.6%, respectively. The mean PTV dose was 70 Gy for both sets of plans for all the 20 patients.

**TABLE 4:** Summary of the dosimetric parameters for the dosimetrically‐ and NTCP‐optimized VMAT plans, for PTV > 300 cm^3^.

Dosimetric parameters	Dosimetric plan	NTCP plan
PTV		
PTV V_95%_ (%)	96.3 ± 1.3	96.2 ± 1.4
Range (%)	94.4–98.7	94.4–98.4
*p*	0.80	
Heart		
Mean heart dose (Gy)	8.8 ± 3.7	11.0 ± 4.6
Range (Gy)	3.6–16.3	4.7–19.7
*p*	<0.001	
V_5_ (%)	40.3 ± 18.3	43.6 ± 18.3
Range (%)	15.4–70.7	15.2–69.9
*p*	0.06	
Left lung		
Mean lung dose (Gy)	25.5 ± 3.4	23.5 ± 3.4
Range (Gy)	19.3–30.8	18.5–29.4
*p*	0.002	
V_20_ (%)	49.5 ± 7.4	43.9 ± 6.6
Range (%)	34.9–58.3	30.9–53.2
*p*‐value	0.006	

**TABLE 5:** Summary of the dosimetric parameters for the dosimetrically‐ and NTCP‐optimized VMAT plans, for PTV < 300 cm^3^.

Dosimetric parameters	Dosimetric plan	NTCP plan
PTV		
PTV V_95%_ (%)	97.6 ± 1.6	97.6 ± 1.8
Range (%)	94.7–99.4	95.0–99.8
*p*	0.83	
Heart		
Mean heart dose (Gy)	8.1 ± 5.4	10.0 ± 6.4
Range (Gy)	2.2–15.5	2.8–18.3
*p*	<0.001	
V_5_ (%)	43.4 ± 32.7	47.1 ± 34.5
Range (%)	6.7–94.4	9.8–95.4
*p*	0.02	
Left lung		
Mean lung dose (Gy)	22.2 ± 6.5	19.2 ± 3.4
Range (Gy)	16.5–40.2	15.4–27.8
*p*	0.02	
V_20_ (%)	43.2 ± 12.5	38.4 ± 9.7
Range (%)	29.5–77.0	37.5–89.3
*p*	<0.001	

In the dosimetrically optimized plans, the average MHD was 8.8 Gy for PTV > 300 cm^3^ and 8.1 Gy for PTV < 300 cm^3^, respectively. In the NTCP‐optimized plans, the average MHD was 11.0 Gy for PTV > 300 cm^3^ and 10.0 Gy for PTV < 300 cm^3^, respectively. After the NTCP‐optimization, in the cases with PTV > 300 cm^3^, MHD had an average increase of 2.2 Gy (from 8.8  to 11.0 Gy with statistical significance, *p* < 0.001), while in the cases with PTV < 300 cm^3^, MHD had an average increase of 1.9 Gy (from 8.1  to 10.0 Gy with statistical significance, *p* < 0.001). Similarly, after the NTCP‐optimization, the heart V_5_ had an average increase of 3.3% (from 40.3% to 43.6%, *p* = 0.06) and an average increase of 3.7% (from 43.4% to 47.1%, *p* = 0.02) in the cases of PTV > 300 and < 300 cm^3^, respectively.

Regarding left lung (minus PTV), in the dosimetrically optimized plans, the average mean doses were 25.5 Gy for PTV > 300 cm^3^ and 22.2 Gy for PTV < 300 cm^3^. In the NTCP‐optimized plans, the average mean doses were 23.5  and 19.2 Gy, respectively. So, after the NTCP‐optimization, the mean dose had an average decrease of 2.0 Gy (from 25.5  to 23.5 Gy with statistical significance, *p = *0.002) and an average decrease of 3.0 Gy (from 22.2  to 19.2 Gy with statistical significance, *p* = 0.02) in the cases of PTV > 300 and < 300 cm^3^, respectively. Additionally, after the NTCP‐optimization, the V_20_ of left lung had an average decrease of 5.6% (from 49.5% to 43.9%, with statistical significance, *p = *0.006) and an average decrease of 4.8% (from 43.2% to 38.4%, with statistical significance, *p <* 0.001) in the cases of PTV > 300 and < 300 cm^3^, respectively.

The MHD constraint was satisfied in both the dosimetrically‐ and NTCP‐optimized sets of plans (average: 8.4 vs. 10.5), whereas the V_20_ constraint for left lung was violated in 7 out of the 40 plans (average: 46.4% vs. 41.1%).

### 3.3 Radiobiological comparison

The results of the plan optimization analysis using radiobiological metrics are presented in Tables 6 and 7. For the patients with PTV > 300 and < 300 cm^3^, the average TCP values were 82.7% and 82.8%, respectively in the dosimetrically‐ and NTCP‐optimized plans.

**TABLE 6:** Summary of the radiobiological evaluation for the dosimetrically‐ and NTCP‐ optimized VMAT plans for PTV > 300 cm^3^.

Radiobiological parameters	Dosimetric plan	NTCP plan
PTV		
TCP (%)	82.7 ± 0.1	82.7 ± 0.1
Range	82.5–82.8	82.4–82.8
*p*	0.32	
*D*_mean_ (Gy)	70.0 ± 0.0	70.0 ± 0.0
Range	0.0–0.0	0.0–0.0
*p*		
Heart		
NTCP (%)	13.7 ± 4.9	17.6 ± 6.8
Range	7.3–23.5	9.5–31.3
*p*	<0.001	
*D*_mean_ (Gy)	8.8 ± 3.7	11.0 ± 4.6
Range	3.6–16.3	4.7–19.7
*p*	<0.001	
Left lung		
NTCP (%)	86.0 ± 9.5	79.5 ± 11.8
Range	64.1–96.6	63.1–95.3
*p*	0.008	
*D*_mean_ (Gy)	25.5 ± 3.4	23.5 ± 3.4
Range	19.3–30.8	18.5–29.4
*p*	0.002	

**TABLE 7:** Summary of the radiobiological evaluation for the dosimetrically‐ and NTCP‐ optimized VMAT plans for PTV < 300 cm^3^.

Radiobiological parameters	Dosimetric plan	NTCP plan
PTV		
TCP (%)	82.8 ± 0.1	82.8 ± 0.0
Range	82.6–82.8	82.7–82.8
*p*	0.45	
*D*_mean_ (Gy)	70.0 ± 0.0	70.0 ± 0.0
Range	0.0–0.0	0.0–0.0
*p*		
Heart		
NTCP (%)	11.5 ± 5.5	14.2 ± 6.7
Range	5.5–20.8	6.4–24.5
*p*	<0.001	
*D*_mean_ (Gy)	8.1 ± 5.4	10.0 ± 6.4
Range	2.2–15.5	2.8–18.3
*p*	<0.001	
Left lung		
NTCP (%)	70.7 ± 14.6	57.9 ± 15.8
Range	46.8–99.5	37.5–89.3
*p*	<0.001	
*D*_mean_ (Gy)	22.2 ± 6.5	19.2 ± 3.4
Range	16.5–40.2	15.4–27.8
*p*	0.02	

For heart, in the dosimetrically optimized plans, the average NTCP value was 13.7% for PTV > 300 cm^3^ and 11.5% for PTV < 300 cm^3^, whereas in the NTCP‐optimized plans, the corresponding values were 17.6% and 14.2%, respectively. Those results indicate an average increase of 3.9% (from 13.7% to 17.6% with statistical significance, *p* < 0.001) for PTV > 300 cm^3^ and an average increase of 2.7% (from 11.5% to 14.2% with statistical significance, *p* < 0.001) for PTV < 300 cm^3^, respectively.

Regarding left lung (minus PTV), in the dosimetrically optimized plans, the average NTCP values were 86.0% for PTV > 300 cm^3^ and 70.7% for PTV < 300 cm^3^, respectively. In the NTCP‐optimized plans, these values were 79.5% and 57.9%, respectively. This means that after the NTCP‐optimization, the lung NTCP value had an average decrease of 6.5% (from 86.0% to 79.5% with statistical significance, *p* = 0.008) in the cases with PTV > 300 cm^3^ while for PTV < 300 cm^3^, the lung NTCP value had an average decrease of 12.8% (from 70.7% to 57.9% with statistical significance, *p* < 0.001).

As it is shown in Table 2, the NTCP model parameters for heart and lung are characterized by uncertainties (confidence intervals). Those uncertainties should be accounted for when comparing the changes in NTCP for those OARs. For this reason, a sensitivity analysis was performed, and the results are shown in the Appendix. Tables A1 and A2 show how those uncertainties propagate in the estimation of the heart and left lung NTCPs and whether those uncertainties would “wash out” any statistically significant differences observed in the NTCP results shown in Tables 6 and 7. The results shown in the Appendix used the highest and lowest values of the confidence intervals of the model parameters and they confirmed the statistical significance of the NTCP differences between the dosimetrically‐ and NTCP‐optimized plans for heart and left lung in all the examined cases.

## 4 DISCUSSION

Lung irradiation has been associated with the development of acute pneumonitis and late fibrosis after radiotherapy. The risk for acute and chronic radiation‐induced lung morbidity has been found to depend on total dose to lung, dose per fraction, and irradiated lung volume.^38^, ^39^ Clinically symptomatic RP (grade ≥ 1) occurs in 83%–100% of lung cancer cases, whereas RP (grade ≥ 2) occurs in up to 10% of patients irradiated for breast cancer and in around 30% of lung cancer cases.^15^, ^17^, ^40^ Modern treatment techniques have contributed to the reduction of the amount of irradiated lung volume. In a recent trial, no case of RP was found in patients who received lung‐V_20_ < 30%.^41^

Although fatal RP appears to be a rare event, higher incidences of grade 5 RP have been recently reported.^17^, ^42^, ^43^ In those cases, large volumes of total lung and contralateral lung receiving 5 Gy were involved and the patients died of pneumonitis 2–5 months after radiotherapy. The addition of low dose constraints (V_5_ < 60%) to the standard V_20_ and mean lung dose seemed to correlate with the risk for grade 5 RP. Nevertheless, until now, no consensus has been reached in the community regarding the optimum dose distribution in the lung (e.g., a small dose to a large volume vs. a large dose to a small volume).

A large meta‐analysis^44^ involving 40 781 patients randomly assigned to radiotherapy versus no radiotherapy showed that heart dose is related to the risk of major coronary events (e.g., death from ischemic heart disease, coronary revascularization, and myocardial infarction), which start appearing within 5 years after radiotherapy.^44^, ^45^, ^46^, ^47^, ^48^ The absolute lung cancer rates for smokers and non‐smokers from modern radiotherapy is estimated to be 4% and 2%, respectively. The corresponding cardiac mortality risks have been estimated to be 0.3% and 0.3%, respectively.^44^ However, milder heart toxicities have been considered lately [e.g., heart valve dysfunction (RVD)] in treatment plan optimization. This radiation‐induced heart complication has an incidence rate of around 26% in the first decade after radiotherapy.

There are many studies that have investigated the validity of different NTCP models or dose‐response relations for RP and heart complications. Niezink et al.^49^ validated the QUANTEC and APPELT models using 612 patients with incidence of RP ≥ grade 2 of 14.5%, accounting for the impact of MLD, age, and smoking status. Similarly, Cella et al.^32^ concluded that the risk of radiation induced valvular disease cannot be modeled using NTCP models based only on heart dose‐volume distribution. The authors stated that a predictive model with an improved performance can be obtained by considering heart and lung volume factors, indicating that heart‐lung interactions are apparently important for the examined endpoint.

Also, Ricardi et al.^50^ derived a dose‐response relation (D_50_ = 22.4 Gy and γ_50_ = 2.2) for radiation‐induced lung injury (RTOG grade 0–1 vs. 2–3) based on MLD for 60 patients treated with SBRT for lung tumors. Kong et al.^51^ studied 109 patients, who reported 14.6% of grade ≥ 2 RP and found that this toxicity was associated with lung‑dosimetric parameters such as the MLD, V_20_ and lung‐NTCP. For V_20_ ≤ 30% and MLD ≤ 20 Gy, the positive and negative predictive values were 50%–71% and 85%–89%, respectively.

In the present study, observable NTCP differences were found only for left lung rather than paired‐lungs. This is due to the low dose received by the contralateral lung in VMAT plans (steep dose gradient). This approach is supported by the work by Agrawal et al.^52^ who used 52 patients of non‑small cell lung cancer and RP was correlated much better with the ipsilateral lung (ipsi‐V_20_, ipsi‐V_5_, and ipsi‐MLD) than the paired‐lungs (V_20_, V_5_, and MLD) dose volume parameters.

In Tables 4 and 5, it is shown that the PTV coverage was the same in the dosimetrically‐ and NTCP‐optimized plans. Furthermore, on average the dose to heart increased (based on the values of MHD and V_5_), whereas the dose to left lung decreased (based on the values of MLD and V_20_). Those changes are graphically illustrated in Figures 1 and 2. In Tables 6 and 7, the TCP values remain unchanged between the dosimetrically‐ and NTCP‐optimized plans as a consequence of maintaining the same coverage. The heart‐NTCP values were also calculated using the model parameters by Eriksson et al.^33^ which correspond to the endpoint of cardiac mortality. In this case, the average NTCP values were around 1%, which is in line with the historical incidence rates. For this endpoint, the NTCP‐optimization had a minimal effect.

**FIGURE 4:**
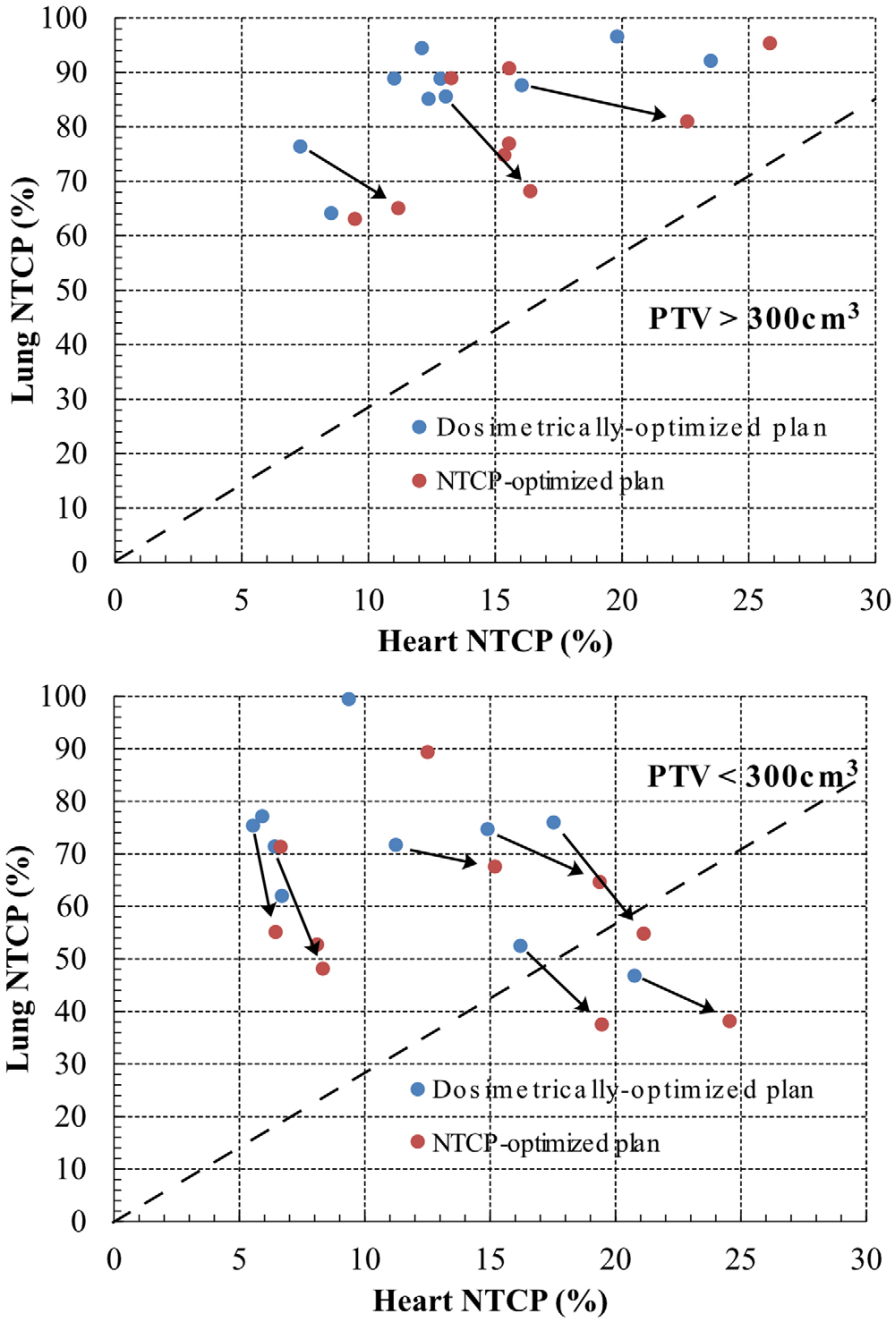
Plot showing the change in the NTCP values of the left lung and heart between the dosimetrically and NTCP‐optimized plans as a function of the PTV size. The dashed lines indicate the ratio of the clinically reported incidence rates for the examined lung and heart complications.

Tables 6 and 7 as well as Figure 4, clearly indicate the unbalanced distribution of the toxicity risks between left lung and heart. For the clinical endpoints examined, the historical incidence rates are ∼85% for grade ≥ 1 RP for lung cancer radiotherapy vs. ∼30% for RVD). Consequently, it would be expected that the data of the patients should fall closer to the dashed diagonal lines in Figure 4. In the dosimetrically‐optimized plans, the NTCP data dominate the upper left corner of the plot, which corresponds to higher lung‐NTCP versus heart‐NTCP values. In the NTCP‐optimized plans, most of the NTCP data have moved close to the diagonal line improving the NTCP ratio between those two organs at risk. However, in certain cases of large PTV sizes located very close or overlapping with heart, the plan re‐optimization using NTCP did not produce considerable different results compared to the dosimetrically‐optimized plans.

The presented work is a treatment plan evaluation and optimization study. It would be ideal if the outcome data of the patients regarding the examined radiation induced cardiac and pulmonary complications were available so that the NTCP predictions of the study could be correlated with those results. The model parameter used for the NTCP calculations of heart and left lung have been derived from clinical trials, which indicates a strong support for the clinical relevance of the presented results and conclusions. However, the best validation of those conclusions would be a direct comparison with the outcome data of the patients, who participated in this study. Regarding other cardiac and pulmonary endpoints such as pericarditis, myocarditis, coronary heart disease, pulmonary edema, and pulmonary fibrosis, those symptoms are associated with different NTCP model parameter values than the ones used in this study. For most of those endpoints, NTCP modeling studies are limited or absent in the literature.

Previous studies^19^, ^20^ have demonstrated the level of impact that different DCAs have on the derivation of NTCP model parameters as well as on the NTCP predictions in lung radiotherapy. For this reason, the scope of this work did not include this type of analysis. Furthermore, substructures of heart such as left and right ventricles, left and right atrium, left anterior descending artery and pericardium could also have been used in the analysis. However, the lack of NTCP model parameters for most of those structures for the examined cardiac endpoint prevented the expansion of this analysis in those directions. So, the study focused on the most common practice regarding the DCA used by the treatment planning system as well as the structures and clinical endpoints.

In this type of study, it is critical to make sure that the model parameters that are used to calculate the NTCP values of lung and heart have been derived by studies using the same clinical endpoints (RP and RVD). It has been demonstrated by many studies that radiobiological measures are better associated with the clinical outcome than solely dosimetric ones.^53^ One of the reasons for their slow clinical implementation is the lack of enough validation studies that would also estimate the level of uncertainty of those TCP and NTCP predictions. Consequently, further randomized trials are needed to determine whether optimizing lung cancer cases in the way it was presented in this work would produce a clinically observable outcome difference compared to the standard dosimetric plan optimization methods. Hopefully, future prospective studies will confirm the good predictive performance and robustness of the presented treatment optimization approach.

## 5 CONCLUSION

Our results indicate that there is a systematic unbalance in the levels of sparing between left lung and heart, which varies based on the PTV size. Improved levels of dose trade‐off could be identified using NTCP metrics, while maintaining the same level of tumor coverage and sparing of other nearby organs at risk.

## AUTHOR CONTRIBUTIONS

Georgios Komisopoulos: Conceptualization; data collection/curation; formal data analysis; investigation; methodology; writing the original draft. Panayiotis Mavroidis: Conceptualization; data collection/curation; formal data analysis; investigation; methodology; correcting‐original draft. Fotini Simopoulou: Verified the analytical methods; contributed to the design and implementation of the research; reviewed and corrected the final manuscript. George Kyrgias: Verified the analytical methods; contributed to the design and implementation of the research; reviewed and corrected the original draft. Kiran Pant: Conceptualization; data collection/curation; formal data analysis; investigation; methodology. Yiannis Roussakis: Verified the analytical methods; contributed to the design and implementation of the research; reviewed and corrected the final manuscript. Kyriaki Theodorou: Supervised the findings of this work; verified the analytical methods; contributed to the design and implementation of the research; reviewed and corrected the final manuscript. All authors discussed the results and contributed to the final manuscript.

## CONFLICT OF INTEREST STATEMENT

The authors declare no conflicts of interest.

## 1

**TABLE A1:** Summary of the radiobiological evaluation for the dosimetrically and NTCP‐optimized VMAT plans for PTV > 300 cm^3^. Those results were produced using the highest and lowest values in the confidence intervals of the model parameters of heart and lung.

Radiobiological parameters	Clinical plan	Re‐optimized plan
Heart_Cella (D_50_ = 48.5 Gy, γ = 0.62, s = 0.001)		
NTCP (%)	1.8 ± 1.0	2.9 ± 1.7
Range	0.8–4.3	1.0–6.6
*p*	0.008	
$\bar{\bar{D}}$ (Gy)	6.6 ± 3.0	9.3 ± 4.0
Range	2.5–12.7	3.5–16.1
*p*	0.004	
Heart_Cella (D_50_ = 22.7 Gy, γ = 0.24, s = 1.0)		
NTCP (%)	26.4 ± 5.5	30.5 ± 7.2
Range	18.9–37.4	21.2–44.5
*p*	<0.001	
$\bar{\bar{D}}$ (Gy)	8.1 ± 3.5	10.6 ± 4.4
Range	3.2–14.9	4.7–19.2
*p*	<0.001	
Left Lung (D_50_ = 15.64 Gy, γ = 1.1, s = 0.05)		
NTCP (%)	83.9 ± 9.9	77.3 ± 12.1
Range	61.5–95.5	60.5–94.0
*p*	0.007	
$\bar{\bar{D}}$ (Gy)	22.8 ± 3.1	21.0 ± 3.3
Range	17.3–28.3	17.1–26.9
*p*	0.002	
Left lung (D_50_ = 15.46 Gy, γ = 1.3, s = 0.05)		
NTCP (%)	87.8 ± 8.9	81.7 ± 11.2
Range	66.9–97.4	65.9–96.4
*p*	0.009	
$\bar{\bar{D}}$ (Gy)	23.0 ± 3.1	21.2 ± 3.3
Range	17.6–28.4	17.5–27.1
*p*	0.002	

**TABLE A2:** Summary of the radiobiological evaluation for the dosimetrically and NTCP‐optimized VMAT plans for PTV < 300 cm^3^. Those results were produced using the highest and lowest values in the confidence intervals of the model parameters of heart and lung.

Radiobiological parameters	Clinical plan	Re‐optimized plan
Heart_Cella (D_50_ = 48.5 Gy, γ = 0.62, s = 0.001)		
NTCP (%)	1.7 ± 1.2	2.4 ± 1.7
Range	0.6–3.7	0.7–5.1
*p*	0.007	
$\bar{\bar{D}}$ (Gy)	5.8 ± 4.0	7.4 ± 4.9
Range	1.4–11.7	1.9–14.1
*p*	<0.001	
Heart_Cella (D_50_ = 22.7 Gy, γ = 0.24, s = 1.0)		
NTCP (%)	24.3 ± 6.9	27.3 ± 8.2
Range	16.7–35.0	17.7–39.1
*p*	<0.001	
$\bar{\bar{D}}$ (Gy)	6.6 ± 4.5	8.5 ± 5.2
Range	1.5–13.5	2.3–15.9
*p*	<0.001	
Left lung (D_50_ = 15.64 Gy, γ = 1.1, s = 0.05)		
NTCP (%)	68.5 ± 14.8	56.0 ± 15.4
Range	45.0–99.2	36.6–87.4
*p*	<0.001	
$\bar{\bar{D}}$ (Gy)	19.8 ± 6.0	16.8 ± 2.7
Range	15.0–36.3	13.9–23.3
*p*	0.03	
Left lung (D_50_ = 15.46 Gy, γ = 1.3, s = 0.05)		
NTCP (%)	73.0 ± 14.2	60.1 ± 16.0
Range	49.0–99.6	38.9–91.0
*p*	<0.001	
$\bar{\bar{D}}$ (Gy)	20.1 ± 5.9	17.1 ± 2.7
Range	15.3–36.3	14.2–23.4
*p*	0.03	
